# The Strength Training Research Paradox: Are We Studying the Right People?

**DOI:** 10.3390/sports14100440

**Published:** 2026-10-08

**Authors:** Jesper Augustsson

**Affiliations:** Department of Sport Science, Linnaeus University, 391 82 Kalmar, Sweden; jesper.augustsson@lnu.se

**Keywords:** resistance training, strength training, athletes, training status, external validity, advanced training methods, strength, hypertrophy

## Abstract

Strength training research has expanded considerably, yet the evidence base remains heavily skewed toward individuals with limited training experience. Recent evidence indicates that only 2.3% of participants included in a sample of 26 highly cited systematic reviews and meta-analyses of core strength training programme variables were classified as highly trained, elite, or world-class athletes, whereas 97.7% were sedentary, untrained, or recreationally trained. This imbalance becomes particularly problematic when evaluating advanced strength training methods. Recent evidence illustrates this problem. A systematic review found broadly similar strength and power adaptations across advanced and traditional resistance training methods, while also identifying some method- and outcome-specific benefits. However, many of the underlying studies included participants with limited training experience, raising questions about the applicability of these findings to highly trained populations. Rather than simply reiterating the limited representation of highly trained athletes, this Perspective distinguishes between two research questions: whether an advanced method can produce adaptation and whether it provides additional benefit when conventional training is already producing limited further adaptation. It argues that training status becomes particularly important to external validity when the hypothesised benefit of a method depends on overcoming diminishing adaptation. Findings from novice and moderately trained individuals cannot be assumed to generalise to highly trained athletes, whose potential for further adaptation and need for more specialised training stimuli may differ substantially. To address this issue, a conceptual framework and practical recommendations are presented to improve the alignment between the training methods under investigation, participant training status, and the populations to which findings are intended to generalise. Without greater consideration of training status, strength training research risks producing conclusions that extend beyond the populations in which the evidence was actually established.

## 1. Introduction

Strength (or resistance) training research encompasses a broad range of programme variables and methods, including training load, volume, frequency, rest intervals, exercise order, movement velocity, periodisation, and exercise selection. Beyond these basic programming variables, more specialised or “advanced” resistance training methods have been developed to manipulate the training stimulus in ways that extend beyond conventional programming approaches. These questions have been examined in numerous experimental studies and subsequently synthesised in systematic reviews and meta-analyses [1]. Collectively, this literature has contributed to the evidence base informing strength training prescription. Coaches and practitioners now have access to an evidence base that would have been difficult to imagine only a few decades ago.

Yet an important question remains: who are we actually studying? This Perspective examines the implications of this question for the alignment between research questions, participant training status, and study design, particularly when advanced training methods are evaluated.

This question is particularly important when research findings are translated from exercise science into recommendations for highly trained athletes. The problem is not that research involving less-trained populations lacks value, but that the applicability of findings depends on whether the participants studied adequately represent the population to which the conclusions are extended.

The magnitude of this problem has recently been illustrated by Fry et al. [2], who examined a sample of 26 highly cited systematic reviews and meta-analyses published between 2010 and 2024 and addressing core strength training programme variables. Across 18,574 participants, only 2.3% were classified as highly trained, elite, or world-class athletes using the participant-classification framework of McKay et al. [3]. Fry et al. selected the 26 reviews using a modified stratification process intended to represent the different resistance-training topics and publication years, while also including highly cited reviews. The imbalance was consistent across programme variables: participants classified below the highly trained level accounted for 90.3–100% of the samples across exercise choice, order, volume, load, rest intervals, frequency, and tempo. Although this estimate characterises the reviews sampled by Fry et al. rather than the strength training literature as a whole, it highlights the limited representation of highly trained athletes within an influential segment of the evidence base.

This discrepancy becomes particularly striking when the research question itself concerns advanced strength training methods. The more specialised the method and the more its rationale depends on overcoming adaptation to conventional training, the more consequential participant training status becomes. A method designed to solve an advanced training problem should, at some point, be tested in people who actually have that problem.

## 2. Advanced Methods—But for Whom?

Cowley et al. [4] recently conducted a systematic review and meta-analysis comparing traditional strength training with seven advanced strength training methods, including approaches such as accentuated eccentric loading, flywheel training, rest redistribution, cluster sets, supersets, and drop sets. Their analysis represents an ambitious attempt to determine whether advanced approaches produce superior adaptations in strength, power, hypertrophy, jumping, and sprinting.

For strength and power outcomes, Cowley et al. [4] found broadly similar adaptations across traditional and advanced resistance training methods, with the credible intervals for relative effects crossing zero. However, the findings were not uniformly null across outcomes and methods. Flywheel training produced superior jump adaptations, with greater benefits observed in shorter interventions and those involving lower volume loads, while network meta-regression suggested that rest redistribution may be more beneficial for strength development in females than in males. Cowley et al. [4] did not report an overall distribution of participants according to training status, precluding a direct quantification of the proportion of highly trained individuals represented in their analysis.

Taken together, these findings illustrate that the effects of advanced methods may depend on the specific method, outcome, and population studied. At the same time, the generally similar strength and power adaptations raise an important question concerning the populations in which advanced methods are evaluated.

Importantly, the label “advanced” does not imply that these methods are necessarily intended only for advanced trainees. Their rationales are heterogeneous: some may be used to manipulate fatigue, maintain performance within a session, improve time efficiency, alter loading characteristics, or provide a different mechanical stimulus. The appropriate population therefore depends on the hypothesised benefit of the method and the research question being addressed. Training status becomes particularly consequential when the proposed benefit specifically concerns overcoming diminishing adaptation to conventional training or providing an additional benefit once substantial adaptation has already occurred.

These considerations also highlight an important distinction in the underlying literature. Demonstrating that an advanced resistance training method can produce adaptation is not necessarily the same as determining whether it provides an additional benefit when conventional training is already producing limited further adaptation. The latter question is particularly relevant when advanced methods are intended to overcome diminishing adaptation with increasing training status [5,6,7]. Consequently, studies in individuals who remain highly responsive to conventional training may provide valid comparisons for that population, but may not adequately test whether advanced methods offer additional benefits in highly trained individuals.

Importantly, the recent ACSM Position Stand [1] provides further context for this question. Its overview of 137 systematic reviews found that relatively few resistance-training prescription variables consistently influenced the primary adaptations, although specific approaches were associated with enhanced outcomes; for example, eccentric contractions/overload were identified as favourable for hypertrophy. At the same time, the authors explicitly noted that most included reviews examined individuals with minimal or no resistance-training experience and that much of the synthesised evidence was derived from inexperienced trainees. Thus, the available evidence does not imply that advanced methods are uniformly ineffective. Rather, their potential value appears to depend on the specific method, outcome, training context, and population in which they are applied.

This distinction is more than semantic. If an intervention is proposed specifically to overcome a plateau, manipulate a stimulus that has become less effective, or provide a marginal advantage when conventional programming already produces only small gains, then studying individuals who are still highly responsive to basic progressive training changes the question being answered. Less-trained individuals typically remain highly responsive to conventional progressive resistance training [5,6,7,8], and a study in this population may therefore validly show that an advanced method provides no additional benefit under the conditions studied. It may say much less about whether that method provides an additional benefit for advanced trainees.

The appropriate conclusion may therefore not be that advanced methods provide no additional benefit in general. Rather, the available evidence may primarily demonstrate that they provide no additional benefit in non-advanced trainees who remain highly responsive to conventional training. Those are not equivalent propositions.

Cowley et al. [4] recognise this limitation themselves and call for future studies involving participants with greater training age and relative strength. Their findings therefore suggest a more fundamental question than whether advanced strength training methods are superior to traditional approaches: “In whom should advanced strength training methods be expected to be superior?” Only then can an experimental population be selected that provides a meaningful test of the hypothesis. The present Perspective extends this recommendation by framing training status as a question-specific component of external validity and by linking population selection to the hypothesised benefit of the method and the study design required to test it.

This does not imply that advanced methods must prove superior in highly trained athletes. Many may not. The point is methodological rather than promotional: claims about the value or lack of value of advanced methods should be based on populations capable of testing the rationale for those methods.

## 3. Training Status Is More than a Participant Characteristic

Training status is frequently reported in strength training studies as one of several descriptive characteristics alongside age, sex, body mass, and training history. However, when studying training adaptations, training status is more than a demographic descriptor. It influences both the magnitude of adaptation that can reasonably be expected and potentially the stimulus required to produce further adaptation.

The principle of diminishing returns is fundamental to training. As early as 1988, Sale [8] noted that individuals with little previous strength training experience generally demonstrate rapid improvements even during relatively short-term strength training programmes or research interventions. Subsequent quantitative evidence supports the importance of training status for the dose–response relationship. Rhea et al. [7], in a meta-analysis of resistance-training studies, reported that the intensity, frequency, and volume associated with maximal strength development differed according to training status, with trained individuals requiring a greater training stimulus than untrained individuals. In athletic populations, Peterson et al. [6] similarly identified dose–response relationships between training prescription and strength development. Wernbom et al. [5], reviewing the effects of resistance-training frequency, intensity, volume, and mode on muscle hypertrophy, likewise demonstrated substantial variation in the training doses associated with adaptation across populations and training conditions. More recently, Lopez et al. [9] reported greater hypertrophic responses in untrained than in recreationally trained individuals and explicitly interpreted this finding in relation to the principle of diminishing returns. Collectively, these findings support the broader principle that the stimulus sufficient to produce adaptation is not independent of previous training exposure. As training experience increases and an individual approaches a higher level of adaptation, further improvements generally become progressively more difficult to achieve. For an elite athlete, an improvement that would appear trivial in a novice population may be practically meaningful; conversely, the large changes routinely observed in inexperienced participants may be unrealistic in an advanced population.

This has consequences for experimental comparisons. If traditional progressive strength training and an advanced method are both applied to individuals who remain highly responsive to systematic training, substantial improvements may occur in both conditions. However, this does not necessarily make a between-method difference more or less detectable. Detectability depends on the magnitude of the true between-method difference relative to within-group variability and measurement error. Training status may influence both components: less-trained individuals may show larger overall adaptations but also substantial interindividual variability, whereas highly trained individuals may show smaller absolute adaptations and smaller between-method differences. The implications of training status for statistical inference therefore depend on the signal-to-noise ratio rather than on the magnitude of adaptation alone. Consequently, when the expected between-method effect is small relative to variability, larger samples are required to achieve adequate statistical power and reduce the risk of Type II error [10,11].

Training status may consequently influence not only the magnitude of an observed effect but also our ability to answer the research question itself.

## 4. What Does “Trained” Actually Mean?

A further difficulty is that the label “trained” is remarkably imprecise. Two participants can both satisfy a criterion such as having performed strength training for one or two years while differing substantially in training quality, consistency, exercise selection, loading history, relative strength, competitive background, and proximity to their performance ceiling. The binary distinction between “trained” and “untrained” can therefore conceal exactly the characteristic that matters most for the research question.

This problem is not unique to strength training research. McKay et al. [3] identified the term “elite” as particularly inconsistently defined across sport science and sports medicine and proposed a six-tier Participant Classification Framework ranging from sedentary (Tier 0) to world-class (Tier 5). Their framework incorporates both training volume and objective performance criteria, illustrating why generic labels such as “trained” or “elite” may provide insufficient information about the actual calibre of research participants.

Training age alone is also insufficient. Five years of structured, progressive strength training under qualified supervision is not equivalent to five years of intermittent recreational gym attendance. Likewise, competitive level alone may be misleading: an elite endurance athlete can be highly trained in a general sporting sense while remaining relatively inexperienced in strength training. The relevant definition of advanced status must therefore be specific to the adaptation and method being investigated.

For studies of advanced strength training methods, a more informative description would combine several indicators: years of systematic strength training, recent training consistency, relative strength or other objective performance criteria, competitive level where relevant, and the nature of the participant’s habitual programme. Cowley et al. [4], for example, discuss >5 years of strength training experience or relative strength criteria such as a back squat of ≥2 times body mass as potentially useful indicators. However, fixed relative-strength thresholds may vary in relevance according to factors such as sex, body mass, sport, and the specific strength demands of that sport, and should therefore not be treated as universal criteria for advanced training status. No single threshold will suit every population, but explicit operationalisation would be a major improvement over the generic label “resistance-trained”.

This is especially important in systematic reviews. If studies using very different definitions of “trained” are pooled, the resulting category may be statistically convenient but physiologically heterogeneous. A review can then appear to address trained individuals while containing relatively few participants who would be regarded by coaches or researchers as genuinely advanced trainees.

The appropriate study population should therefore be defined in relation to the research question rather than by a generic classification of training status. General athletic calibre and method- or adaptation-specific training status should be treated as related but distinct characteristics. An athlete may be elite within a sport while having limited experience with the specific form of strength training under investigation. Conversely, substantial strength-training experience does not necessarily imply elite sporting calibre. Importantly, an untrained population may be entirely appropriate when aligned with the research question. For example, Hubal et al. [12] deliberately recruited individuals without recent resistance-training experience in a large multicentre study of variability in strength and hypertrophic responses to a standardised training programme. For research evaluating advanced strength training methods, participant classification should therefore describe not only general athletic calibre but also the training status specifically relevant to the intervention and adaptation being studied. Table 1 operationalises this distinction by linking the type of research question to the relevant training status, the minimum participant information required to evaluate population–question alignment, and key methodological considerations.

## 5. A Large Evidence Base with a Small Elite Foundation

The findings of Fry et al. [2] place this issue in a broader context. Within their sample of 26 highly cited systematic reviews and meta-analyses of core strength training programme variables, highly trained, elite, and world-class athletes represented only 2.3% of participants. Thus, an extensive literature can provide considerable statistical precision while still offering limited direct evidence for populations that are poorly represented within it. The problem is not that research involving untrained participants lacks value. Such research is essential for understanding fundamental adaptations to resistance exercise and for informing exercise prescription for the general population. Nor should every strength training question be studied in elite athletes. For many questions, recruiting novices is entirely appropriate. The problem arises when conclusions derived primarily from these populations migrate into recommendations concerning advanced athletes without sufficient acknowledgement of the limits to external validity.

This issue is particularly relevant for systematic reviews and meta-analyses. Increasing the number of studies and participants improves statistical precision, but not necessarily applicability to populations that are largely absent from the underlying studies. The evidence examined by Fry et al. [2] provides a concrete example: across 18,574 participants in 26 highly cited systematic reviews and meta-analyses, only 2.3% were classified as highly trained, elite, or world-class. A large evidence base can therefore provide considerable statistical precision while still offering limited direct evidence for a poorly represented target population. Practitioners working with highly trained athletes should not ignore evidence from less-trained populations, but neither should they assume direct transferability.

## 6. The Absence-of-Evidence Problem

Care is also required when interpreting null findings. If advanced and traditional strength training produce similar adaptations in novice or moderately trained individuals, the appropriate inference is restricted to those populations. It cannot establish equivalence among highly trained athletes unless highly trained athletes have actually been studied.

Put simply: evidence that advanced methods provide no additional benefit in non-advanced trainees is not evidence that they provide no additional benefit in advanced trainees. Nor does the opposite necessarily follow. Although Cowley et al. [4] identified some method- and outcome-specific benefits, there remains insufficient evidence to determine whether advanced methods provide superior adaptations specifically in highly trained athletes.

This distinction is crucial because a Perspective arguing for better population alignment could otherwise be misread as an argument in favour of advanced methods themselves. The argument is not that advanced strength training methods are effective, and science has failed to recognise their superiority, but rather that the relevant experiments have too rarely been performed.

A related paradox emerges when researchers do succeed in recruiting well-trained participants. Their inclusion improves the relevance of the study, but simultaneously makes the research question more difficult to answer. As training status increases and the magnitude of further adaptation becomes smaller, relatively small samples may provide limited ability to distinguish potentially meaningful differences between training strategies.

The study by Schoenfeld et al. [18] provides a useful illustration. Twenty well-trained men were allocated to strength training programmes in which each muscle group was trained either once or three times per week, with only 9–10 participants ultimately completing each condition. The participants had a mean resistance-training experience of 4.5 ± 3.1 years. For bench press strength, the higher-frequency group improved by 10.2 kg (10.6%), compared with 6.3 kg (6.8%) in the lower-frequency group. The adjusted between-group estimate was β = 9.87 (*p* = 0.14); a confidence interval for this estimate was not reported. The authors themselves acknowledged that the small sample size and substantial interindividual variability limited the ability to detect differences between conditions. A complementary example is provided by Angleri et al. [19], who compared drop-set and crescent-pyramid training with traditional resistance training in well-trained men using a within-subject design. Participants had 6.4 ± 2.0 years of resistance-training experience and were required to squat at least 130% of body mass. At the group level, the training protocols produced similar adaptations in muscle hypertrophy and strength, while individual-response analyses likewise showed similar hypertrophic responses for the vast majority of participants.

This illustrates the other side of the strength training research paradox. The more highly trained the participants, the more relevant they may become to questions of training optimisation, but the smaller the expected adaptations and potentially the greater the challenge of adequately powering a conventional parallel-group intervention study. Simply replacing untrained participants with well-trained participants while retaining small experimental groups may therefore exchange a problem of external validity for one of statistical inference. Importantly, the numerical difference reported by Schoenfeld et al. [18] should not itself be interpreted as evidence that higher training frequency was superior. Rather, the study illustrates why “no significant difference” in a small sample of well-trained participants cannot necessarily be interpreted as evidence that two training strategies are equivalent.

There is also an important difference between “no statistically significant difference”, “evidence of no meaningful difference”, and “insufficient evidence to determine whether a meaningful difference exists”. This distinction can be formalised by defining an a priori smallest effect size of interest (SESOI) and using equivalence testing, such as the two one-sided tests (TOST) procedure, when the research question concerns whether differences are sufficiently small to be considered practically unimportant [13,14]. Bayesian approaches using informative priors can provide a complementary framework by formally incorporating relevant previous evidence into the analysis of small elite-athlete samples [15]. These interpretations become particularly important in studies of highly trained athletes, where sample sizes are often small and expected improvements modest. A non-significant result in a small study provides limited evidence that two interventions are genuinely equivalent. By contrast, a precisely estimated trivial effect with narrow confidence intervals would provide much stronger evidence that any true between-group difference is likely to be small.

Accordingly, researchers and reviewers should resist turning population-specific null findings into universal statements about a method. Conclusions should remain proportional to the participants actually studied.

## 7. From Improvement to Optimisation

The distinction between improvement and optimisation is well established in strength and conditioning and periodisation literature [20]. Here, this distinction is applied specifically to the alignment between research questions and participant training status. For an untrained individual, the central question is often whether a programme produces a meaningful adaptation. For a highly trained athlete, the question is more demanding: among several already effective options, can one strategy produce a small additional benefit, preserve adaptation while reducing fatigue, or solve a specific constraint in the athlete’s programme?

These questions can involve different outcomes, acceptable trade-offs, and thresholds for practical relevance. An advanced athlete may value a small improvement in maximal strength or power if it can be obtained without disrupting sport practice, whereas a method producing similar adaptation at greater complexity or recovery cost may have little practical value. Research intended to inform advanced practice therefore needs to study the stage of the training process at which optimisation, rather than simple responsiveness, is the problem.

## 8. Where Should Strength Training Research Go Next?

Recruiting highly trained and elite athletes is difficult. Samples are smaller, training cannot always be manipulated freely, competition schedules interfere with study designs, and coaches and athletes may resist randomisation to potentially suboptimal conditions. Fry et al. [2] highlight several of these challenges. These practical constraints likely contribute to the predominance of less-trained participants in the existing evidence base.

Nevertheless, methodological convenience should not determine the population to which findings are ultimately applied. If the applied claim concerns highly trained athletes, the difficulty of studying them is a limitation to be addressed, not a reason to assume that evidence from a more accessible population is interchangeable.

Future research evaluating advanced strength training methods should therefore place greater emphasis on aligning the population with the underlying research question. As a practical minimum reporting set, studies addressing advanced strength training questions should, where relevant, report: (1) years of systematic strength training; (2) recent training consistency and history; (3) relative strength or other objective performance criteria relevant to the research question; (4) competitive level; (5) the nature of the participant’s habitual strength training programme; and (6) an explicit justification of why the selected sample is appropriate for the intervention and adaptation being investigated. This information would allow authors, reviewers, and readers to judge whether participants are sufficiently aligned with the research question rather than relying on generic labels such as “trained” or “elite”. Definitions will inevitably differ between sports and research questions, and no single criterion can provide a universal definition of advanced training status. The goal should instead be to make the relationship between the research question and participant characteristics explicit and defensible.

Alternative research designs may also be necessary. Studies involving highly trained and elite athletes may rarely achieve the sample sizes typical of general-population exercise trials. However, studies must also be adequately designed and powered to detect the small but potentially meaningful effects relevant to advanced athletes [10,11]. Importantly, what constitutes a meaningful effect should be specified a priori rather than determined by statistical significance alone. For optimisation research in highly trained athletes, this may involve defining a smallest effect size of interest (SESOI) based on the magnitude of change considered practically relevant in the specific performance context. Equivalence or non-inferiority approaches can then distinguish between insufficient evidence of a difference and evidence that any difference is too small to be practically important [14]. This distinction is particularly relevant when conventional null-hypothesis testing is applied to the small samples typical of elite sport.

Bayesian approaches provide another potentially useful strategy. Informative priors can incorporate relevant previous evidence into analyses of necessarily small elite-athlete samples, thereby allowing existing knowledge to contribute formally to inference rather than treating each small study in isolation [15]. Such priors must, however, be chosen cautiously when previous evidence derives from populations with substantially different training status, since inappropriate borrowing of information could reproduce the same external-validity problem that the present Perspective seeks to address.

Finally, when sufficiently large group studies are unrealistic, replicated single-case or N-of-1 designs may provide a complementary approach for investigating practically meaningful responses in elite athletes [16,17]. These designs do not eliminate problems of measurement error, carryover, motor learning, or within-athlete variability, but they can shift the emphasis from inadequately powered between-group comparisons towards repeated, athlete-specific observations under carefully controlled conditions.

**Figure 1:**
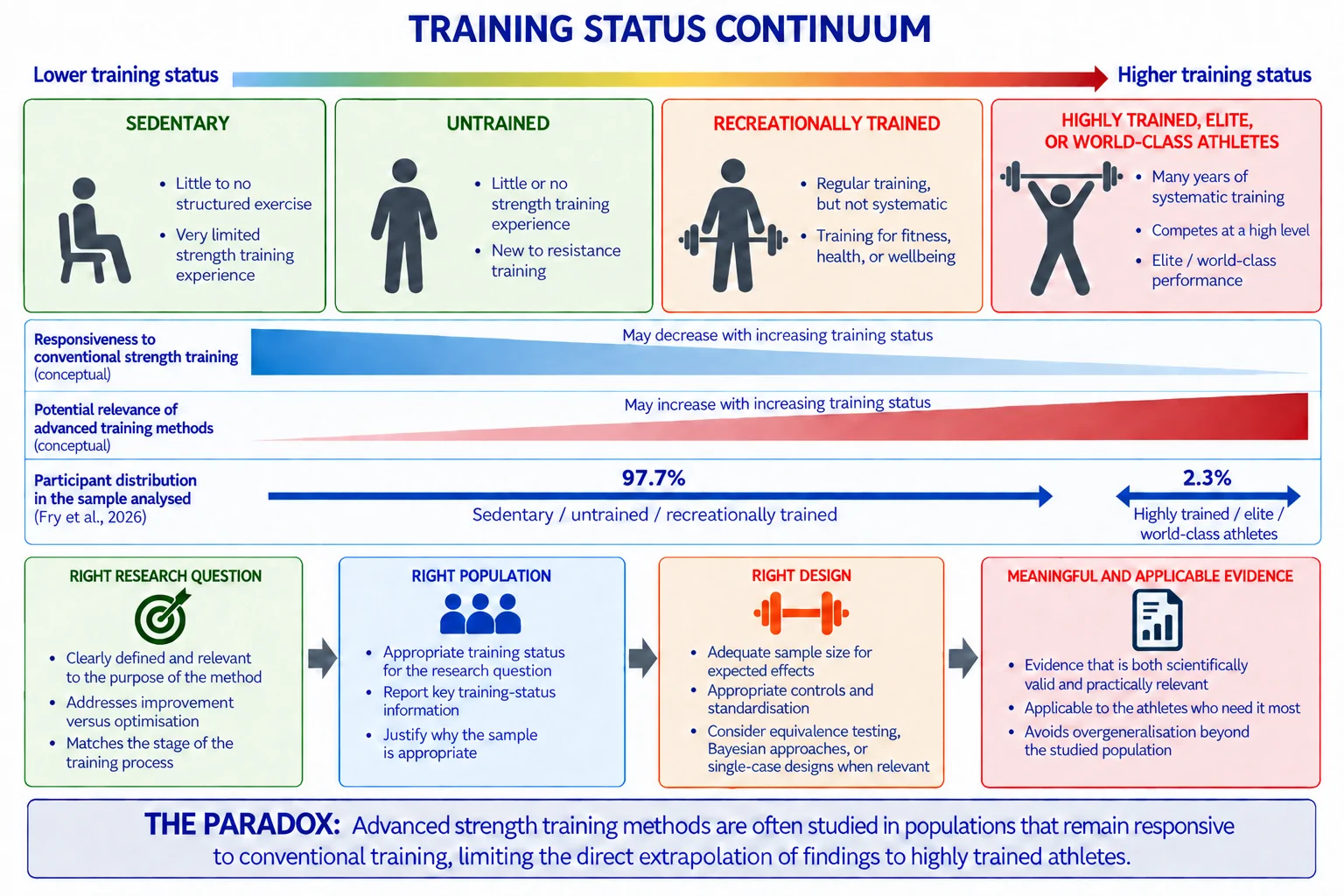
Conceptual representation of the strength training research paradox. As training status increases, responsiveness to conventional strength training may decrease, while the potential relevance of advanced training methods may increase. These gradients are conceptual rather than empirically quantified relationships. In the sample of 26 highly cited systematic reviews and meta-analyses examined by Fry et al. [2], highly trained, elite, and world-class athletes represented 2.3% of participants, compared with 97.7% classified as sedentary, untrained, or recreationally trained. The figure illustrates the need to align the research question, study population, and research design.

Greater use of multicentre collaboration could increase recruitment while preserving the target population. Repeated-measures and carefully controlled within-subject designs may be useful where appropriate, and longer observation periods may sometimes be necessary because further adaptations in advanced athletes may occur more slowly. Individual-response analyses can complement group means when heterogeneity itself is practically important, although such analyses must be interpreted cautiously and with appropriate attention to measurement error [21]. Taken together, these considerations highlight the need to align the research question, study population, and research design when investigating strength training adaptations, as illustrated in the conceptual framework proposed in this Perspective (Figure 1).

In some contexts, pragmatic designs embedded within real training environments may provide greater ecological validity than highly standardised interventions. However, this creates an inherent trade-off: greater ecological validity may come at the cost of increased uncontrolled variability, making the small effects relevant to highly trained athletes more difficult to detect. Conversely, highly standardised designs may improve experimental control while reducing the extent to which the intervention reflects elite training practice. The appropriate balance between experimental control and ecological validity should therefore be determined by the specific research question. Excluding elite athletes because their training is difficult to standardise risks producing a highly controlled literature with limited relevance to the population of interest.

Systematic reviews can contribute by stratifying evidence according to training status rather than treating it solely as a secondary moderator when data permit. At minimum, reviewers should make the distribution of training status transparent and ensure that conclusions do not extend beyond the populations represented. A large body of evidence dominated by untrained and recreationally trained participants should be described as such. Such stratification, however, depends on adequate reporting in the primary studies. Poor or inconsistent reporting of training history, recent training exposure, relative strength, and competitive level may make meaningful classification impossible at the review stage. Greater standardisation of training-status reporting in primary resistance-training studies, potentially incorporated alongside established reporting frameworks such as CONSORT and CERT [22,23], would therefore improve both the interpretability of individual studies and the ability of subsequent reviews to evaluate population-specific evidence.

Most importantly, researchers should distinguish between questions concerning whether a method can produce adaptation and questions concerning whether a method provides an additional advantage once conventional training is no longer sufficient. These are fundamentally different questions and may require fundamentally different participants.

## 9. Conclusions

Strength training science has generated an impressive volume of research, but volume of evidence should not be confused with breadth of applicability. Fry et al. [2] have recently highlighted the limited representation of highly trained athletes within a sample of highly cited systematic reviews and meta-analyses of core strength training programme variables. Cowley et al. [4] bring the issue into particularly sharp focus by showing that much of the research evaluating advanced strength training methods has involved relatively untrained populations.

Their findings should not be dismissed. On the contrary, they provide valuable evidence that advanced methods may be unnecessary for individuals who continue to respond well to conventional strength training, at least when the proposed advantage concerns enhancing further adaptation.

But a different question remains largely unanswered: do advanced strength training methods provide additional benefits for advanced trainees? At present, the most scientifically defensible answer may simply be: we do not know, because we have rarely tested them in that population.

The broader lesson applies to strength training research as a whole. Participant training status should be considered part of the research question itself whenever the expected effect depends on previous adaptation. However, selecting the appropriate population is only part of the solution; studies must also be adequately designed to detect the small but potentially meaningful effects relevant to advanced athletes. The paradox is therefore twofold: we often ask advanced training questions of non-advanced trainees, yet when we study advanced trainees, conventional small-sample designs may struggle to provide clear answers. Addressing this problem requires explicit alignment between the research question, participant training status, and study design, together with transparent reporting of the characteristics that determine whether the sample is appropriate for the question being asked. Future strength training research should therefore define the target population in relation to the research question, report the training-status characteristics required to evaluate that alignment, and use study designs capable of detecting the magnitude of effect that is practically relevant in that population.

## Figures and Tables

**Table 1:** Conceptual alignment between strength training research questions, participant training status, and methodological considerations.

Research Question	Relevant Training Status/Tier	Minimum Training-Status Information	Methodological Considerations
Does ST produce an adaptation?	Untrained/novice for the adaptation studied	Previous ST exposure; recent training history	Conventional group designs generally feasible; relatively large adaptations expected
Does manipulation of a basic programme variable improve outcomes?	Appropriately trained for the programme variable and adaptation studied	Training age; recent training consistency; habitual programme; relevant performance criteria	Population-specific inference; define practically meaningful effects where appropriate
Does an advanced method provide additional benefit over conventional ST?	Appropriately trained in relation to the method and adaptation studied	Training age; recent consistency; habitual programme; relative strength or other objective criteria; competitive level where relevant	Small expected between-method effects; define SESOI a priori; consider equivalence testing and within-subject designs [10,11,13,14]
Does a method optimise elite performance?	Elite competitive level and advanced method-/adaptation-specific training status	All relevant training-status descriptors plus explicit justification of sample–question alignment	Small samples and effects; consider Bayesian approaches, replicated single-case/N-of-1 designs, multicentre approaches, and the trade-off between experimental control and ecological validity [12,15,16,17]

ST = Strength training.

## Data Availability

No new data were created or analyzed in this study. Data sharing is not applicable to this article.
